# Reply to Marino et al. Choroidal Thickness Measurements in the Case of Diabetic Macular Edema. Comment on “Amjad et al. Choroidal Thickness in Different Patterns of Diabetic Macular Edema. *J. Clin. Med.* 2022, *11*, 6169”

**DOI:** 10.3390/jcm12082877

**Published:** 2023-04-14

**Authors:** Rida Amjad, Cheong-Ah Lee, Hafiz Muhammad Umer Farooqi, Hina Khan, Dong-Guk Paeng

**Affiliations:** 1 Department of Retina, Amanat Eye Hospital, Rawalpindi 46000, Pakistan; 2 Ocean & Biomedical Ultrasound Laboratory, Department of Ocean System Engineering, Jeju National University, Jeju 63243, Republic of Korea

We are pleased to see that Marino et al. have written a Comment: “Choroidal Thickness Measurements in the Case of Diabetic Macular Edema” [1] in response to our paper published in the Journal of Clinical Medicine by Amjad et al. [2].

The authors are thankful for the careful reading and for giving valuable opinions and feedback on our reach work.

First, we accept that choroidal thickness changes with axial length, but as already mentioned in the exclusion criteria, we excluded the eyes exhibiting high refractive errors so that the axial length of these eyes may not affect our results. We did not consider that as a confounder within the set limits of refractive error due to the small sample size. However, this may be a confounder below these limits, and we accept this as a limitation of our study.

Second, Heidelberg Eye Explorer software (Version 1.10.2.0) was used to measure choroidal thickness in the central and two parafoveal points on the horizontal SD-OCT scan. The scans had recognizable foveal depression. Moreover, we opted for two methods with respect to choroidal thickness measurements (i.e., manual and automated image processing) for the sake of accuracy. As mentioned in the choroidal thickness measurement criteria, we also measured parafoveal ChT along with the central ChT and calculated mean values for choroidal thickness to avoid bias. Furthermore, ChT evaluation was performed on SD-OCT scans (as per availability), and the subfoveal ChT was measured using a caliper tool (the unit of measurement is microns) in the OCT Heidelberg Eye Explorer software. The vertical distance was measured at the fovea from the hyperreflective line of Bruch’s membrane to the hyperreflective line of the chorio–scleral interface, i.e., a line perpendicular to the RPE–Bruch complex.

Finally, we took a consecutive population of healthy individuals. One of them had one eye lost to trauma. Two of them had eyes with poor picture quality because of cataracts, and since there was no retinal abnormality, they were still included in our healthy patient population. While being analyzed retrospectively, we excluded the three above-mentioned eyes of the healthy individuals.

## Data Availability

The data presented in the study are available upon reasonable request from the corresponding authors.
